# Effect of Ethanolic Extract of *Embelia Ribes* on Dyslipidemia in Diabetic Rats

**DOI:** 10.1080/15604280214278

**Published:** 2002

**Authors:** Uma Bhandari, Raman Kanojia, K. K. Pillai

**Affiliations:** 1 Department of Pharmacology Faculty of Pharmacy Hamdard University New Delhi 110062 India

## Abstract

Diabetes mellitus has been treated orally with herbal remedies based on folk medicine since ancient times. *Embelia ribes burm* (Myrsinaceae), known commonly as vidanga, was used in Ayurveda for its anthelmintic activity. Ayurveda describes vidanga as pungent, causes increase in digestive fire, and cures flatulence and colic. A single study reported the antihyperglycemic activity of decoction of *E. ribes* in glucose-induced hyperglycemic albino rabbits. In the present study, the lipid-lowering and antioxidant potential of ethanolic extract of *E. ribes burm* was investigated in streptozotocin (40 mg/kg, IV, single injection)-induced diabetes in rats. Twenty days of orally feeding the extract (200 mg/kg) to diabetic rats resulted in significant (*P* < 0.01) decrease in blood glucose, serum total cholesterol, and triglycerides, and increase in HDLcholesterol levels when compared to pathogenic diabetic rats. Further, the extract also lowered the liver and pancreas thiobarbituric acid–reactive substances (TBARSs) values (*P* < 0.01) when compared to TBARS values of liver and pancreas of pathogenic diabetic rats. The results of test drug were comparable to gliclazide (25 mg/kg, orally), a standard antihyperglycemic agent. This is the first pilot study to provide biochemical evidence of potential of *E. ribes* in diabetic dyslipidemia.


*Int. Jnl. Experimental Diab. Res.*, 3:159–162, 2002
Copyright © 2002 Taylor & Francis
1560-4284/02 $12.00 + .00
DOI: 10.1080/15604280290013856

Taylor & Francis healthsciences

**Research**

### Effect of Ethanolic Extract of *Embelia ribes* on Dyslipidemia in Diabetic Rats

**Uma Bhandari, Raman Kanojia, and K. K. Pillai**

*Department of Pharmacology, Faculty of Pharmacy, Hamdard University, New Delhi, India*

**Diabetes mellitus has been treated orally with herbal remedies based on folk medicine since ancient times. *Embelia ribes burm* (Myrsinaceae), known commonly as vidanga, was used in Ayurveda for its anthelmintic activity. Ayurveda describes vidanga as pungent, causes increase in digestive fire, and cures flatulence and colic. A single study reported the antihyperglycemic activity of decoction of *E. ribes* in glucose-induced hyperglycemic albino rabbits. In the present study, the lipid-lowering and antioxidant potential of ethanolic extract of *E. ribes burm* was investigated in streptozotocin (40 mg/kg, IV, single injection)-induced diabetes in rats. Twenty days of orally feeding the extract (200 mg/kg) to diabetic rats resulted in significant ($P < 0.01$) decrease in blood glucose, serum total cholesterol, and triglycerides, and increase in HDL-cholesterol levels when compared to pathogenic diabetic rats. Further, the extract also lowered the liver and pancreas thiobarbituric acid–reactive substances (TBARSs) values ($P < 0.01$) when compared to TBARS values of liver and pancreas of pathogenic diabetic rats. The results of test drug were comparable to gliclazide (25 mg/kg, orally), a standard antihyperglycemic agent. This is the first pilot study to provide biochemical evidence of potential of *E. ribes* in diabetic dyslipidemia.**

**Keywords** Diabetes; Dyslipidemia; *Embelia ribes*; Lipid Peroxidation; Streptozotocin

According to the Framingham Heart Study [1], dyslipidemia, which can range from hypercholesterolemia to hyperlipoproteinemia, is one of the many modifiable risk factors for coronary artery disease (CAD), stroke, and peripheral vascular disease. In diabetic dyslipidemia, lipid abnormalities may be the result of unbalanced metabolic states of diabetes (i.e., hyperglycemia and insulin resistance).

Received 14 February 2002; accepted 11 April 2002.
Address correspondence to Uma Bhandari, Department of Pharmacology, Faculty of Pharmacy, Hamdard University, New Delhi 110062, India.

Improved control of hyperglycemia does moderate diabetes-associated dyslipidemia; therefore, lipid-modifying treatment is warranted in many diabetic patients. There is also considerable evidence that oxidative damage is increased in diabetes, though the mechanisms are not clear [2, 3].

Efforts continue in the field of medicine to find insulin substitutes from synthetic or plant sources for the treatment of diabetes. In traditional medicine, several medicinal plants or their extracts have been used to treat diabetes [4].

*Embelia ribes burm* (family, Myrsinaceae), known commonly as vidanga, is used in Ayurveda as anthelmintic [5]. Ayurveda describes vidanga as pungent, causes increase in digestive fire, and cures flatulence and colic. One Ayurvedic formulation, vidangadya curna (powder of vidanga), containing vidanga as main ingredient is taken with honey to alleviate obesity [6]. In a preliminary study, Tripathi [7] reported the antihyperglycemic activity of decoction of the *E. ribes* fruits in glucose-fed albino rabbits. The present study was undertaken to investigate the effect of ethanolic extract of *E. ribes* on diabetic dyslipidemia induced by streptozotocin (STZ) in wistar rats.

### MATERIALS AND METHODS

### Preparation of the Extract

Dried *E. ribes* fruits, 200 g, were purchased locally from a grocery shop in New Delhi, India (in India, it is commonly available) and authenticated by a pharmacognosist, Prof. Mohd. Ali in our institute. A voucher specimen was retained in the department (UB # 04).

The fruits were soxhlet extracted with 90% ethanol in a soxhlet apparatus for 72 hours. The solvent was removed under reduced pressure to give a dry extract, 7% yield *w/w* (with respect to the crude material) and dose equivalent to 200 mg of the crude drug per kilogram body weight was calculated, and suspended in 2% *v/v* Tween 80 solution for the experiment.

### Experimental Induction of Diabetes in Rats

Experiments on animals were conducted after obtaining approval from Hamdard University Animal Ethics Committee, which is registered with Committee for the Purpose of Control and Supervision of Experiments on Animals (CPCSEA), Government of India, India (Registration no. 173/CPCSEA, dated 28 January, 2000).

Wistar rats of either sex (150 to 200 g) were obtained from the central animal house facility of Hamdard University of Delhi. They were acclimatized in an air-conditioned room at 22°C ± 2°C for 7 days and provided with free access to food (Gold Mohur rat pellet diet, Lipton India, Bangalore, India) and water.

After fasting for 18 hours, the rats were injected intravenously through tail vein with a single dose of 40 mg/kg STZ (Sigma, St. Louis, MO, USA), freshly dissolved in citrate buffer (pH 4.5). After injection, the rats had free access to food and water and were given 5% glucose solution to drink overnight to counter hypoglycemic shock. Diabetes in rats was identified by moderate polydipsia and marked polyuria.

After 3 days, the fasting blood glucose levels were determined by *ortho*-toluidine method [8, 9]. The rats showing fasting blood glucose more than 200 mg/100 dL were considered diabetic and selected for the experimentation [10, 11].

### Experimental Procedure

Normal and diabetic rats (n = 10 each) were randomly divided into 4 groups of 10 rats each: Group I, normal healthy control; group II, pathogenic diabetic control (STZ treated only); group III, STZ + ethanolic *E. ribes* extract treated (200 mg/kg); group IV, STZ + gliclazide treated (25 mg/kg), a standard control. The test and standard drug were fed orally for 20 days. Groups I and II rats received 2% Tween 80 solution orally once a day for 20 days.

### Blood Collection and Biochemical Estimations

On the 21st day, fasting blood samples were collected from tail vein of all the groups of rats. Whole blood was collected for estimation of blood glucose [8, 9]. Serum was separated for the estimation of total serum cholesterol [12], high-density lipoprotein (HDL)-cholesterol [13], and triglycerides [14].

### Measurement of Tissue Lipid Peroxidation

On the evening of the 21st day, all fasted rats were killed by decapitation under light ether anaesthesia. Liver and pancreas were removed immediately and washed with ice-cold normal saline. Ten-percent homogenate of above tissues were prepared separately at 10,000 rpm in cooling centrifuge. Supernatant thus obtained was used for measurement of thiobarbituric acid–reactive substances (TBARSs), which can be measured by the formation of malondialdehyde (MDA) after the breakdown of polyunsaturated fatty acids [15]. Liver and pancreas protein contents were evaluated by the method of Lowry using bovine serum albumin (BSA) as standard [16].

### Statistical Analysis

The results are presented as mean ± SEM using 1-way analysis of variance test (ANOVA) followed by Dunnett's *t* test. $P < 0.01$ was considered significant.

### RESULTS

The mean blood glucose levels in rats fed on normal diet (group I) alone was stable throughout the experimental period. Conversely, in the STZ-treated group, group II, there was significant rise in blood glucose level, as compared to group I. Drug treatment in STZ-treated rats for 20 days significantly reduced ($P < 0.01$) blood glucose levels in groups III and IV when compared to pathogenic group II rats (Table 1).

The ethanolic extract of *E. ribes* also significantly ($P < 0.01$) reduced serum total cholesterol and triglycerides and increased

**TABLE 1**
Effect of ethanolic extract of *E. ribes* on STZ-diabetic rats

| Groups | Blood glucose (mg/dl) | Serum total cholesterol (mg/dl) | Serum HDL cholesterol (mg/dl) | Serum triglycerides (mg/dl) |
|---|---|---|---|---|
| I Normal control | 92.1 ± 6.0 | 69.1 ± 3.4 | 45.7 ± 1.4 | 51.9 ± 3.9 |
| II STZ (40 mg/kg, IV) | 573.9 ± 29.6* | 101.1 ± 2.47* | 33.2 ± 4.39* | 123.1 ± 4.5* |
| III STZ + Ethanolic *E. ribes* extract (200 mg/kg, PO) | 243.9 ± 41.3# | 81.5 ± 1.25# | 69.2 ± 4.0# | 56.9 ± 4.9# |
| IV STZ + gliclazide (25 mg/kg/day, PO) | 248.1 ± 25.8# | 87.2 ± 1.5# | 53.9 ± 3.7# | 67.0 ± 3.0# |

*Note.* Values are mean ± SEM (n = 10).
*$P < 0.01$ when compared with group I; #$P < 0.01$ when compared with group II.

**TABLE 2**
Effect of ethanolic extract of *E. ribes* on tissues lipid peroxidation in STZ-diabetic rats

| Groups | Lipid peroxides (mol/mg protein) in liver | Lipid peroxides (mol/mg protein) in pancreas |
|---|---|---|
| I Normal control | 0.0441 ± 0.008 | 0.0303 ± 0.008 |
| II STZ diabetic control (40 mg/kg, IV) | 0.1861 ± 0.007* | 0.2019 ± 0.010* |
| III STZ + ethanolic *E. ribes* extract (200 mg/kg) | 0.0798 ± 0.001[#] | 0.0883 ± 0.006[#] |
| IV STZ + gliclazide (25 mg/kg) | 0.0635 ± 0.001[#] | 0.0811 ± 0.006[#] |

*Note.* Values are mean ± SEM (n = 10).
*$P < 0.01$ when compared with group I; [#]$P < 0.01$ when compared with group II.

the HDL-cholesterol levels as compared to pathogenic diabetic rats, i.e., Group II. Furthermore, the results of the test drug were comparable to gliclazide, a standard antihyperglycemic agent. There was no significant change in food consumption during the administration of ethanolic extract of *E. ribes* in dose of 200 mg/kg. However, the experimental animals showed marked polyuria and moderate polydipsia.

STZ treatment also induced a statistically significant increase in liver and pancreas lipid peroxide levels ($P < 0.01$) as compared to group I. *E. ribes* and gliclazide treatments lowered the liver and pancreas TBARS values ($P < 0.01$) as compared to group II diabetic rats (Table 2).

### DISCUSSION

Cardiovascular diseases constitute the main cause of morbidity and mortality in diabetes mellitus. Diabetic individuals have a 2- to 4-fold increased risk of clinical atherosclerotic disease [17]. Dyslipidemia has been proven to be the most important modifiable risk factor contributing to atherosclerosis in diabetes [18]. Furthermore, there is widespread acceptance of a possible role for reactive oxygen species, generated as a result of hyperglycemia, in causing many of the secondary complications of diabetes, such as nephropathy, retinopathy, and neuropathy [19].

The inability of the modern synthetic approach to provide a satisfactory answer has led to a shift in focus to alternative forms of therapy based on drugs derived from plants. The present study was an effort to investigate the effect of ethanolic extract of *E. ribes* on diabetic dyslipidemia induced by STZ in rats. The study revealed the significant antihyperglycemic activity ($P < 0.01$) of ethanolic extract of *E. ribes*. Furthermore, extract treatment also produced significant fall in serum total cholesterol and triglyceride levels, indicating profound lipid-lowering activity of the test drug. The study also indicated the presence of antioxidant principles in the extract.

In conclusion, the present study shows that increased oxidative stress is apparent in STZ-induced diabetic animals. The ethanolic extract of *E. ribes* can protect tissues from lipid peroxidation. The extract also exhibits a significant lipid-lowering activity in these rats. Further studies are being undertaken to explain more fully the mechanism(s) of the lipid-lowering and antioxidant effects of *E. ribes*.

### REFERENCES

[1] Chong, P. H., and Bachenheimer, B. S. (2000) Current, new and future treatments in dyslipidemia and atherosclerosis. *Drugs,* **60,** 55–93.

[2] Tatsuki, R., Satoh, K., Yamamoto, A., Hoshi, K., and Ichihara, K. (1997) Lipid peroxidation in the pancreas and other organs in streptozotocin diabetic rats. *Jpn. J. Pharmacol.,* **75,** 267–273.

[3] West, I. C. (2000) Radicals and oxidative stress in diabetes. *Diabetic Med.,* **17,** 171–180.

[4] Akhtar, F. M., and Ali, M. R. (1984) Study of the antidiabetic effect of a compound medicinal plant prescription in normal and diabetic rabbits. *J. Pak. Med. Assoc.,* **34,** 239–244.

[5] Pandey, V. N. (1996) In: *Pharmacological Investigation of Certain Medicinal Plants and Compound Formulations Used in Ayurveda and Siddha*, 1st ed. pp. 370–376. New Delhi, India: Central Council for Research in Ayurveda and Siddha, Yugantar Press.

[6] Vaidya Arya, M. R. (1999) Sthaulya Cikitsa (Treatment of obesity). In: *A Compendium of Ayurvedic Medicine, Principles and Practice*, pp. 335–339. Delhi, India: Sri Satguru Publications. A division of Indian Book Centre.

[7] Tripathi, S. N. (1979) Screening of hypoglycaemic action in certain indigenous drugs. *J. Res. Indian Med. Yoga & Homeopathy,* **14,** 159–169.

[8] Hultman, E. (1959). Rapid specific method for determination of aldosaccharides in body fluid. *Nature,* **183,** 108–109.

[9] Hyavarina, A., and Nikkita, E. (1962) Specific determination of blood glucose with orthotoluidine. *Clin. Chim. Acta,* **7,** 140–143.

[10] Chempakam, B. (1993) Hypoglycaemic activity of arecoline in betel nut, *Areca catechu* L. *Indian J. Exp. Biol.,* **31,** 474–475.

[11] Zhang, X. F., and Tan, B. K. H. (2000) Antihyperglycaemic and antioxidant properties of Andrographis paniculata in normal and diabetic rats. *Clin. Exp. Pharmacol. Physiol.,* **27,** 358–363.

[12] Demacher, P. N. M., and Hijamaus, A. G. M. (1980) A study of the use of polyethylene glycol in estimating cholesterol. *Clin. Chem.*, **26,** 1775–1778.

[13] Warnick, G. R., Nguye, T., and Albers, A. A. (1951) A comparison of improved precipitation method for quantification of HDL-cholesterol. *J. Biol. Chem.*, **193,** 265–267.

[14] Fossati, P., and Prencipe, L. (1982) Triglyceride estimations. *Clin. Chem.*, **28,** 2077–2080.

[15] Ohkawa, M., Ohishi, N., and Yagi, K. (1979) Assay for lipid peroxides in animal tissue by thiobarbituric acid reaction. *Anal. Biochem.*, **95,** 351–358.

[16] Lowery, O. H., Rosenbrough, M. S., Farr, A. L., and Raudall, R. J. (1951) Protein measurement with the folin phenol reagent. *J. Biol. Chem.*, **193,** 265–267.

[17] Nathan, D. M., Meigs, J., and Sireger, D. E. (1997) The epidemiology of cardiovascular disease in type 2 diabetes mellitus: How sweet is ...... or is it? *Lancet*, **350,** 514–519.

[18] Neimeijer-kanters, S. D. J. M., Banga, J. D., and Erkelens, D. W. (2001) Lipid lowering therapy in diabetes mellitus. *Neth. J. Med.*, **58,** S14–S19.

[19] Gingliano, D., Ceriello, A., and Paolisso, G. (1996) Oxidative stress and diabetic vascular complications. *Diabetes Care*, **19,** 257–267.

